# Key influences on dysglycemia across Fujian’s urban-rural divide

**DOI:** 10.1371/journal.pone.0308073

**Published:** 2024-07-31

**Authors:** LiHan Lin, XiangJu Hu, XiaoYang Liu, GuoPeng Hu

**Affiliations:** 1 College of Physical Education, Huaqiao University, Quanzhou, China; 2 School of Public Health, Fujian Medical University, Fuzhou, China; 3 Department for Chronic and Noncommunicable Disease Control and Prevention, Fujian Provincial Center for Disease Control and Prevention, Fuzhou, Fujian, China; Bangladesh University of Health Sciences, BANGLADESH

## Abstract

**Background:**

Screening and treatment of dysglycemia (prediabetes and diabetes) represent significant challenges in advancing the Healthy China initiative. Identifying the crucial factors contributing to dysglycemia in urban-rural areas is essential for the implementation of targeted, precise interventions.

**Methods:**

Data for 26,157 adults in Fujian Province, China, were collected using the Social Factors Special Survey Form through a multi-stage random sampling method, wherein 18 variables contributing to dysglycemia were analyzed with logistic regression and the random forest model.

**Objective:**

Investigating urban-rural differences and critical factors in dysglycemia prevalence in Fujian, China, with the simultaneous development of separate predictive models for urban and rural areas.

**Result:**

The detection rate of dysglycemia among adults was 35.26%, with rates of 34.1% in urban areas and 35.8% in rural areas. Common factors influencing dysglycemia included education, age, BMI, hypertension, and dyslipidemia. For rural residents, higher income (OR = 0.80, 95% CI [0.74, 0.87]), average sleep quality (OR = 0.89, 95% CI [0.80, 0.99]), good sleep quality (OR = 0.89, 95% CI [0.80, 1.00]), and high physical activity (PA) (OR = 0.87, 95% CI [0.79, 0.96]) emerged as protective factors. Conversely, a daily sleep duration over 8 hours (OR = 1.46, 95% CI [1.03, 1.28]) and middle income (OR = 1.12, 95% CI [1.03, 1.22]) were specific risk factors. In urban areas, being male (OR = 1.14, 95% CI [1.02, 1.26]), cohabitation (OR = 1.18, 95% CI [1.02, 1.37]), and central obesity (OR = 1.35, 95% CI [1.19, 1.53]) were identified as unique risk factors. Using logistic regression outcomes, a random forest model was developed to predict dysglycemia, achieving accuracies of 75.35% (rural) and 76.95% (urban) with ROC areas of 0.77 (rural) and 0.75 (urban).

**Conclusion:**

This study identifies key factors affecting dysglycemia in urban and rural Fujian residents, including common factors such as education, age, BMI, hypertension, and dyslipidemia. Notably, rural-specific protective factors are higher income and good sleep quality, while urban-specific risk factors include being male and central obesity. These findings support the development of targeted prevention and intervention strategies for dysglycemia, tailored to the unique characteristics of urban and rural populations.

## Introduction

Dysglycemia, which includes prediabetes and diabetes, represents a growing global health challenge with significant public health implications [1–3]. Prediabetes is an early stage characterized by higher-than-normal blood glucose levels, which increases the risk of developing type 2 diabetes (T2DM) [4]. Without timely intervention, prediabetes can progress to diabetes, a condition characterized by elevated blood glucose due to insufficient insulin production or ineffective insulin use, leading to complications such as cardiovascular disease, kidney failure, and neuropathy [5–7]. Key factors influencing dysglycemia include genetic predisposition, individual health, lifestyle habits, environmental factors, and social security [8–13].

In China, the largest developing country, the dual urban-rural economic structure has led to significant disparities in healthcare services, social security, and resource allocation between urban and rural areas, affecting income levels, educational attainment, and lifestyles [14–16]. These disparities contribute to different risks of chronic diseases, including T2DM. For instance, higher prevalence rates of T2DM in rural areas compared to urban areas in the United States are attributed to the greater commonality of poverty, obesity, and smoking in these rural settings. [17].

Recent advancements in medical science and artificial intelligence have made machine learning a vital tool for analyzing multidimensional medical data. Machine learning techniques, such as Logistic Regression (LR) and the Random Forest (RF) algorithm, are valuable for predicting and explaining health outcomes and assisting in developing prevention strategies and clinical treatment plans [18,19]. LR provides quantitative interpretations of significant factors through odds ratios (ORs), while the RF algorithm, known for its resilience to noise and reduced risk of overfitting, effectively handles complex data environments [20,21]. These methods are widely recognized for their applications in disease prediction and risk factor assessment [22,23].

Given the distinct social structures and historical contexts across nations, understanding dysglycemia susceptibility among urban and rural populations is essential, particularly in China’s pronounced urban-rural divide. This study uses face-to-face survey data from Fujian, China, and applies univariate analysis, logistic regression (LR) analysis, and random forest (RF) models to identify critical determinants of dysglycemia prevalence. Initially, 18 variables were considered: demographic, social security, lifestyle, and physiological health. Through rigorous univariate and LR analysis, key determinants were identified: common factors such as education, age, BMI, hypertension, and dyslipidemia; rural-specific protective factors like higher income, good sleep quality, and high physical activity; and urban-specific risk factors including being male, cohabitation, and central obesity. By developing a random forest model based on these determinants, the study aims to improve screening efficiency for at-risk individuals. By developing a random forest model based on these determinants, the study aims to improve screening efficiency for at-risk individuals. This research highlights the differences in dysglycemia risk factors between urban and rural areas and proposes targeted, evidence-based strategies for prevention and intervention.

## Methodology

### Study design and setting

This study was based on the Chinese Adults Noncommunicable Disease and Nutrition Surveillance (Fujian segment), a cross-sectional study investigating health-related behaviors and dysglycemia among adults in Fujian Province, China. The baseline dataset used in this study was collected from June 2019 to March 2021.

Considering the geographical, economic, and population characteristics of Fujian Province, a multi-stage cluster random sampling method was employed. In the first phase, 16 districts and counties were selected from among the 86 in Fujian Province using a probability proportionate to size sampling method. In the second stage, within each selected district or county, 6 townships (or streets) were randomly selected using the same method. In the third stage, within each selected township (or street), 3 village committees (or communities) were chosen using simple random sampling, with each having at least 100 households. In the fourth and final stage, within each selected household, 1 individual was surveyed, with the sample size calculated using the Kish Leslie formula [24]. The survey targeted permanent residents of Fujian Province who had lived in the survey areas for 6 months or more, were aged 18 years or older, and excluded pregnant women and individuals with cognitive or language impairments.

A standardized Social Factors Special Survey Form (SFSSF-2019) was utilized, combining face-to-face questionnaire interviews with medical examinations. Uniformly calibrated instruments were employed for physical and laboratory examinations to measure blood pressure, blood lipids, height, and weight, as referenced in studies such as [25–28]. Such as, blood pressure was measured using the Omron HBP-1300 electronic blood pressure monitor on the right upper arm [29]. Measurements were taken three times in a resting state, with intervals of more than five minutes between each measurement. Blood glucose levels were determined using fasting plasma glucose (FPG) [30] and a 2-hour post-75 g oral glucose tolerance test (OGTT) [31] venous blood samples (participants with a history of diabetes did not undergo the glucose challenge).

### Dependent variable

The diagnosis of dysglycemia was defined according to the American Diabetes Association (ADA) [4], diabetes was defined as FPG > = 7.0 mmol/L and/or OGTT > = 11.1 mmol/L. Prediabetes was defined as FPG > = 5.6 mmol/L and < = 6.9 mmol/L, OGTT > = 7.8 mmol/L and < 11.0 mmol/L.

### Independent variable

Combining literature analysis and data, the study classified the 18 variables influencing dysglycemia into 4 categories: demographic variables, social security variables, lifestyle variables, and physiological health variables.

Demographic variables encompassed gender, age, place of residence, level of education, Body Mass Index (BMI), annual income, and marital status, while social security variables included health insurance coverage. The rural and urban residence indicates household living regions and is defined by the National Bureau of Statistics of the People’s Republic of China [32]. Urban areas include cities and towns, that have higher population density, diverse economic activities, and better access to services, while rural areas refer to regions outside of the designated urban boundaries, and are characterized by lower population density, primary reliance on agriculture, and limited access to healthcare. Regarding the socioeconomic background, compared to other countries, China has a large urban-rural disparity in terms of economic income [33]. The categorization of annual income levels was based on the distribution of per capita disposable income for 2023 as reported by the National Bureau of Statistics of China, with classifications into three tiers: Lower income (Under 20,442 yuan, about 2,815 USD), Middle income (Between 20,442 yuan to 50220 yuan, 2,815–6,916 USD) and Higher income = 2(over 50,220 yuan, about 6,916 USD). Physiological health variables consisted of self-rated health status, dyslipidemia, hypertension, central obesity, and chronic diseases. Central obesity was defined according to the "Guidelines for the Prevention and Control of Overweight and Obesity in Chinese Adults," with a waist circumference ≥ 90 cm for men or ≥ 85 cm for women indicating central obesity [34]. The definition of chronic disease incidence included the presence of coronary heart disease, malignant tumors, chronic digestive system diseases, neck and lumbar diseases, chronic obstructive pulmonary disease (COPD), osteoarthritis, cerebrovascular disease, and chronic urinary system diseases, among eight types of chronic conditions. Lifestyle variables were measured by daily sleep duration, sleep quality, self-rated sleep quality, sedentary behavior, PA levels and frequency of alcohol consumption. Sleep duration was categorized into three types according to the recommendations of the American Academy of Sleep Medicine: insufficient sleep (t < 6 hours/day), appropriate sleep (6 hours/day ≤ t < 8 hours/day), and excessive sleep (t ≥ 8 hours/day) [35]. We assessed participants’ sleep quality over the past month using the Pittsburgh Sleep Quality Index (PSQI) [36]. The PSQI scores range from 0 to 21, with higher scores indicating poorer sleep quality. For analysis, we categorized the PSQI scores into three groups: Bad for PSQI scores greater than 10, Average for PSQI scores between 5 and 10, and Good for PSQI scores of 5 or less [37,38]. PA levels were classified into low, moderate, and high based on the scoring rules of the International Physical Activity Questionnaire (IPAQ) Short Form [39]. Sedentary behavior (SB) is defined as an average of more than 6 hours per day spent sitting or lying down, excluding time spent sleeping at night [40]. The remaining independent variables are shown in Table 1 below.

**Table 1:** Variable assignment.

Variable type	Variable name	Variable assignment
**Dependent variable**	Dysglycemia	No Dysglycemia = 0, Dysglycemia = 1
**Demographic variable**	Age	under 65 years old = 0, 65 and older = 1
	Body mass index	underweight = 0 (BMI < 18.5 kg/m2), normal weight = 1 (18.5 < = BMI < 24 kg/m2), overweight = 2 (24 < = BMI < 28 kg/m2) and obese = 3 (BMI > = 28 kg/m2).
	Residence	Rural area = 0, Urban area = 1
	Gender	Female = 0, Male = 1
	Education	Below primary school = 0, Primary school = 1, Junior high school = 2, Junior college and above = 3
	Annual income	Lower income = 0 (under 20442 yuan); Middle income = 1(20442 < = income < = 50220); Higher income = 2(over 50220 yuan)
	Marital status	Solitary = 0, Cohabitation = 1
**Social security variable**	Medical insurance	None = 0, Yes = 1
**Lifestyle variable**	Daily sleep duration	t < 6 h/d = 0, 6 h/d< = t < 8 h/d = 1, t > = 8 h/d = 2
	Sleep quality	Bad = 0, Average = 1, Good = 2
	Sedentary behavior	None = 0, Yes = 1
	PA	Low PA = 0, Moderate PA = 1, High PA = 2
	Alcohol consumption frequency	No drinking = 0, Less than once a month = 1, More than once a month = 2
**Physiological health variable**	Chronic disease	None = 0, Yes = 1
	Hypertension	None = 0, Yes = 1
	Self-rated health	Bad = 0, Average = 1, Good = 2
	Dyslipidemia	None = 0, Yes = 1
	Central obesity	None = 0, Yes = 1

### Data processing

**Fig 1:**
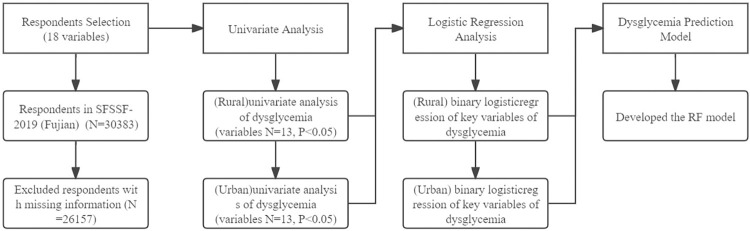
The flow chart of this study.

Logistic regression analysis was used to identify factors influencing dysglycemia, and a predictive model was constructed using the random forest algorithm. Data preprocessing and the construction of the RF model were completed in Python 3.9, using Sklearn, NumPy, Matplotlib, and Pandas. Univariate analysis was conducted using the Chi-square test, while multivariate analysis employed LR statistics, with the significance level set at <0.05. This part of the work was performed in SPSS 26. For a detailed research flowchart, refer to Fig 1 below.

### Ethics

This research is a branch of the Chinese Adults Noncommunicable Disease and Nutrition Surveillance project, conducted within Fujian Province, China, and led by the National Center for Chronic and Noncommunicable Disease Control and Prevention, Chinese Center for Disease Control and Prevention. The project received approval from its ethics committee (#201819). Adhering strictly to ethical standards, we ensured that all participants were fully informed of the study’s purpose, procedures, potential risks, and benefits prior to their participation, and had signed a written informed consent form.

## Results

### Urban-rural differences in variables

Upon exclusion of outliers and missing data, information from 26,157 participants (mean age 53.77, SD ± 14.41) was analyzed. Among these respondents, a significant proportion of rural participants had not pursued education beyond primary school (63.0%). The prevalence of overweight and obesity among urban respondents was 46.1%, higher than the 40.5% observed in rural respondents. Furthermore, a significant fraction of urban respondents, representing 66.8%, belonged to the low-income tier. Dysglycemia was diagnosed in 9,224 participants, yielding a prevalence rate of 35.56%, with 6,567 cases in rural areas and 2,657 in urban areas. These findings are elaborated in Table 2.

**Table 2:** Descriptive statistics.

Variable	Classification	Rural (N = 18359)	Urban (N = 7798)	Total (N = 26157)
**Hyperglycemia**	None	11792 (64.2)	5141 (65.9)	16933 (64.7)
	Yes	6567 (35.8)	2657 (34.1)	9224 (35.3)
**Age**	Under 65	13888 (75.6)	6071 (77.9)	19959 (76.3)
	65 and older	4471 (24.4)	1727 (22.1)	6198 (23.7)
**Body mass index**	Underweight	793 (4.3)	352 (4.5)	1145 (4.4)
	Normal weight	10123 (55.1)	3855 (49.4)	13978 (53.4)
	Overweight	5792 (31.5)	2741 (35.2)	8533 (32.6)
	Obese	1651 (9.0)	850 (10.9)	2501 (9.6)
**Gender**	Female	10111 (55.1)	4321 (55.4)	14432 (55.2)
	Male	8248 (44.9)	3477 (44.6)	11725 (44.8)
**Education**	Below primary school	8381 (45.7)	2745 (35.2)	11126 (42.5)
	Primary school	3185 (17.3)	1241 (15.9)	4426 (16.9)
	Junior high school	3965 (21.6)	1612 (20.7)	5577 (21.3)
	Junior college and above	2827 (15.4)	2200 (28.2)	5027 (19.2)
**Annual income**	Lower income	10964 (59.7)	5208 (66.8)	16172 (61.8)
	Middle income	3404 (18.5)	1054 (13.5)	4458 (17.0)
	Higher income	3991 (21.7)	1536 (19.7)	5527 (21.1)
**Marital status**	Solitary	2472 (13.5)	1157 (14.8)	3629 (13.9)
	Cohabitation	15887 (86.5)	6641 (85.2)	22528 (86.1)
**Medical insurance**	None	69 (0.4)	45 (0.6)	114 (0.4)
	Yes	18290 (99.6)	7753 (99.4)	26043 (99.6)
**Daily sleep duration**	Under 6h	2373 (12.9)	956 (12.3)	3329 (12.7)
	6h-8h	8008 (43.6)	3580 (45.9)	11588 (44.3)
	Over 8h	7978 (43.5)	3262 (41.8)	11240 (43.0)
**Sleep quality**	Bad	2583 (14.1)	1116 (14.3)	3699 (14.1)
	Average	6108 (33.3)	2732 (35.0)	8840 (33.8)
	Good	9668 (52.7)	3950 (50.7)	13618 (52.1)
**Sedentary behavior**	None	12975 (70.7)	5604 (71.9)	18579 (71.0)
	Yes	5384 (29.3)	2194 (28.1)	7578 (29.0)
**PA**	Low PA	12336 (67.2)	5322 (68.2)	17658 (67.5)
	Moderate PA	3866 (21.1)	1821 (23.4)	5687 (21.7)
	High PA	2157 (11.7)	655 (8.4)	2812 (10.8)
**Alcohol consumption frequency**	No drinking	11988 (65.3)	4699 (60.3)	16687 (63.8)
	Less than once a month	1493 (8.1)	832 (10.7)	2325 (8.9)
	More than once a month	4878 (26.6)	2267 (29.1)	7145 (27.3)
**Chronic disease**	None	10505 (57.2)	4450 (57.1)	14955 (57.2)
	Yes	7854 (42.8)	3348 (42.9)	11202 (42.8)
**Self-rated health**	Bad	1651 (9.0)	672 (8.6)	2323 (8.9)
	Average	8644 (47.1)	3448 (44.2)	12092 (46.2)
	Good	8064 (43.9)	3678 (47.2)	11742 (44.9)
**Hypertension**	None	12907 (70.3)	5777 (74.1)	18684 (71.4)
	Yes	5452 (29.7)	2021 (25.9)	7473 (28.6)
**Dyslipidemia**	None	10769 (58.7)	4970 (63.7)	15739 (60.2)
	Yes	7590 (41.3)	2828 (36.3)	10418 (39.8)
**Central obesity**	None	13387 (72.9)	5067 (65.0)	18454 (70.6)
	Yes	4972 (27.1)	2731 (35.0)	7703 (29.4)

### Univariate analysis of factors influencing dysglycemia in urban-rural adults

Among 26,157 urban and rural residents in Fujian Province, the prevalence rates of dysglycemia were 34.1% and 35.8%, respectively, with rural residents exhibiting a higher rate than their urban counterparts (χ^2^ = 6.905, P<0.01). Significant differences were observed in urban residents across 13 factors (P<0.05), including age, BMI, gender, education, marital status, daily sleep duration, SB, PA, chronic disease, self-rated health, hypertension, dyslipidemia, and central obesity. Similarly, rural elders showed significant variances in depressive symptoms across 13 factors (P<0.05), specifically age, BMI, education, annual income, marital status, daily sleep duration, sleep quality, PA, chronic disease, self-rated health, hypertension, dyslipidemia, and central obesity, as detailed in **Table 3.**

**Table 3:** Univariate analysis of variables influencing dysglycemia in urban-rural adults.

Variable	Classification	DG (rural%)	χ2	DG (urban%)	χ2
**Age**	Under 65	4614(33.2)	161.022***	1912(31.5)	81.158***
	65 and older	1953(43.7)		745(43.1)	
**Body mass index**	Underweight	235(29.6)	211.252***	81(23.0)	178.020***
	Normal weight	3220(31.8)		1093(28.4)	
	Overweight	2365(40.8)		1079(39.4)	
	Obese	747(45.2)		404(47.5)	
**Gender**	Female	3673(36.3)	3.037*	1404(32.5)	11.353***
	Male	2894(35.1)		1253(36.0)	
**Education**	Below primary school	3429(40.9)	209.627***	1047(38.1)	74.295***
	Primary school	1050(33.0)		470(37.9)	
	Junior high school	1321(27.1)		541(33.6)	
	Junior college and above	767(35.8)		599(27.2)	
**Annual income**	Lower income	4068(37.1)	100.999***	1784(34.3)	1.777
	Middle income	1334(39.2)		370(35.1)	
	Higher income	1165(29.2)		503(32.7)	
**Marital status**	Solitary	944(38.2)	7.268***	356(30.8)	6.601**
	Cohabitation	5623(35.4)		2301(34.6)	
**Medical insurance**	None	23(33.3)	0.179	12(26.7)	1.105
	Yes	6544(35.8)		2645(34.1)	
**Daily sleep duration**	Under 6h	966(40.7)	69.389***	364(38.1)	9.246**
	6h-8h	2613(32.6)		1176(32.8)	
	Over 8h	2988(37.5)		1117(34.2)	
**Sleep quality**	Bad	1060(41.0)	43.351***	380(34.1)	0.012
	Average	2210(36.2)		933(34.2)	
	Good	3297(34.1)		1344(34.0)	
**Sedentary behavior**	None	4606(35.5)	1.413	1858(33.2)	7.472***
	Yes	1961(36.4)		799(36.4)	
**PA**	Low PA	4406(35.7)	17.221***	1821(34.2)	8.946**
	Moderate PA	1461(37.8)		584(32.1)	
	High PA	700(32.5)		252(38.5)	
**Alcohol consumption frequency**	No drinking	4303(35.9)	1.405	1608(34.2)	0.346
	Less than once a month	513(34.4)		276(33.2)	
	More than once a month	1751(35.9)		773(34.1)	
**Chronic disease**	None	3462(33.0)	84.645***	1460(32.8)	7.370***
	Yes	3105(39.5)		1197(35.8)	
**Self-rated health**	Bad	697(42.2)	127.413***	241(35.9)	8.341**
	Average	3338(38.6)		1223(35.5)	
	Good	2532(31.4)		1193(32.4)	
**Hypertension**	None	4056(31.4)	357.161***	1720(29.8)	183.444***
	Yes	2511(46.1)		937(46.4)	
**Dyslipidemia**	None	3257(30.2)	346.179***	1434(35.8)	166.220***
	Yes	3310(43.6)		1223(43.2)	
**Central obesity**	None	4484(33.5)	111.329***	1448(28.6)	194.535***
	Yes	2083(41.9)		1209(44.3)	

DG = Dysglycemia

* Indicates P < 0.1

** Indicates P < 0.05

*** Indicates P < 0.01.

### Logistic regression analysis of key factors of dysglycemia

The results indicate that higher education background is significantly associated with lower odds of dysglycemia in both urban and rural areas. Specifically, in rural areas, higher education is associated with reduced odds of dysglycemia, with odds ratios (OR) as follows: primary school (OR = 0.782, 95% CI = 0.714–0.855), junior high school (OR = 0.868, 95% CI = 0.796–0.946), and junior college and above (OR = 0.765, 95% CI = 0.680–0.848). In urban areas, the OR for individuals with junior college education and above is 0.815 (95% CI = 0.708–0.939).

Additionally, common factors associated with higher odds of dysglycemia in urban and rural residents include BMI (overweight: OR = 1.486, 95% CI = 1.127–1.957; obese: OR = 1.800, 95% CI = 1.322–2.453), age (65 and older: OR = 1.372, 95% CI = 1.211–1.555), hypertension (OR = 1.515, 95% CI = 1.353–1.697), and dyslipidemia (OR = 1.570, 95% CI = 1.419–1.736).

For rural residents, higher income, sleep quality, and PA are associated with lower odds of dysglycemia. Specifically, an annual income exceeding 50,220 yuan (OR = 0.799, 95% CI = 0.735–0.870), average sleep quality (OR = 0.892, 95% CI = 0.802–0.993), good sleep quality (OR = 0.894, 95% CI = 0.801–0.997), and high PA (OR = 0.871, 95% CI = 0.788–0.963) are associated with reduced odds of dysglycemia. Conversely, a daily sleep duration over 8 hours (OR = 1.146, 95% CI = 1.025–1.280), middle income (OR = 1.122, 95% CI = 1.033–1.219), and moderate PA (OR = 1.095, 95% CI = 1.013–1.183) are associated with higher odds of dysglycemia in this demographic.

In urban populations, being male (OR = 1.135, 95% CI = 1.024–1.258), cohabitation marital status (OR = 1.132, 95% CI = 1.022–1.366), and central obesity (OR = 1.351, 95% CI = 1.192–1.530) are associated with higher odds of dysglycemia.

### Urban-rural dysglycemia prediction model

#### Random forest model

The RF model is an ensemble learning algorithm comprising multiple decision trees. It utilizes the bagging algorithm for random sampling of the dataset to generate multiple training sets. Each training set is analyzed using a decision tree as the base classifier, with the final prediction being the outcome of a majority vote across multiple trees [21].

#### Model construction

Employing the Random Forest model, an important analysis was conducted on factors affecting dysglycemia in rural and urban residents, followed by the construction of predictive models. The samples were divided into training and testing sets in a 7:3 ratio, with Rural (N = 18359) and Urban (N = 7798) cohorts. Risk factors identified through logistic regression analysis served as training variables, including Age, BMI, Annual income, Daily sleep duration, PA, Hypertension, and Dyslipidemia for the Rural area, and Age, Gender, BMI, Marital status, Hypertension, Dyslipidemia, and Central obesity for the Urban area.

**Fig 2:**
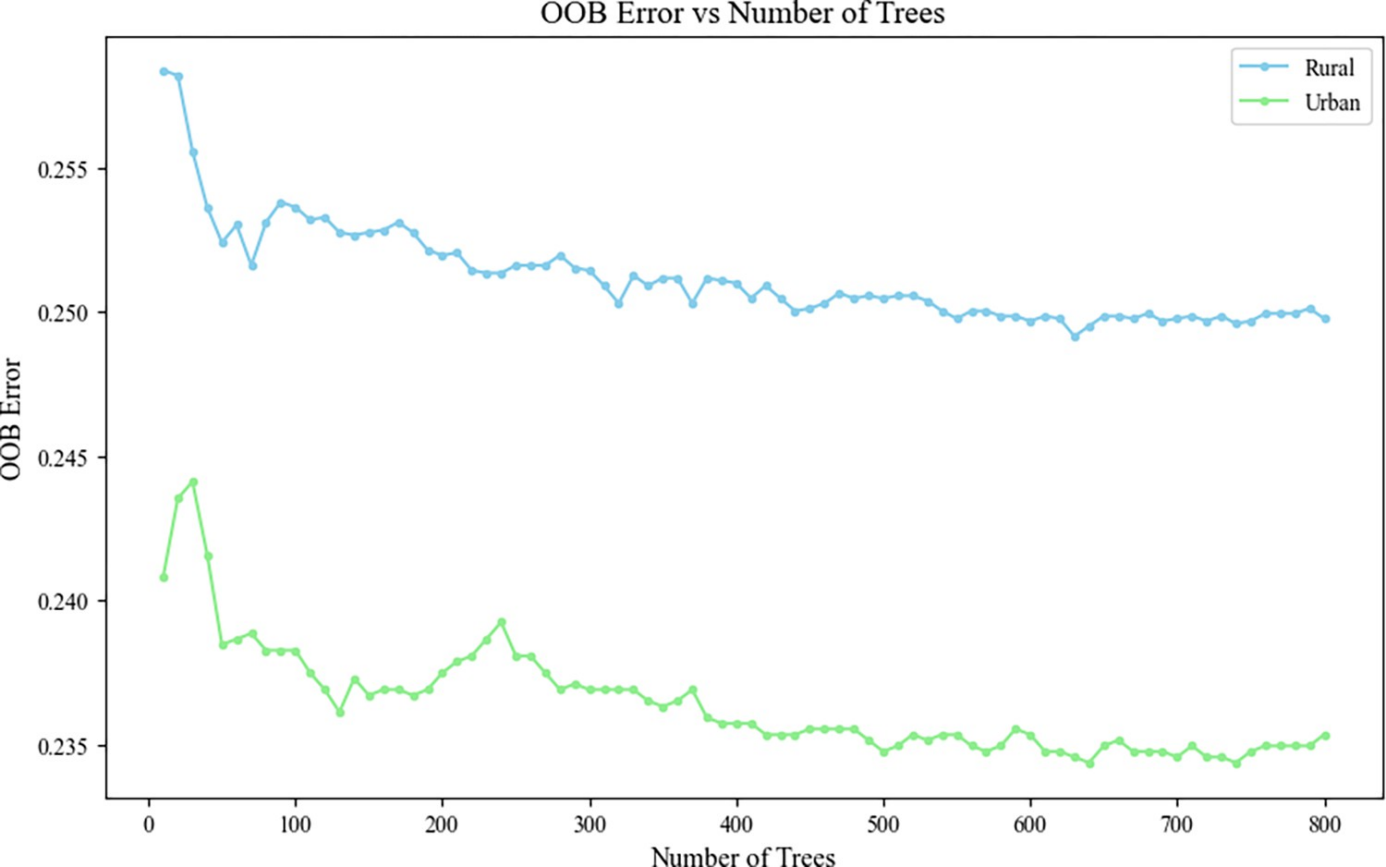
OOB error as the tree number in RF model for rural -urban adults.

Grid search was also utilized to determine the optimal number of decision trees, with Out-of-Bag (OOB) error trends visualized as the number of trees varied. The OOB error, an unbiased estimate of the model’s generalization capability, facilitated the identification of the decision tree count that minimizes OOB, thus determining the optimal parameters for the RF model. The findings are illustrated in Fig 2.

### Results analysis

**Fig 3:**
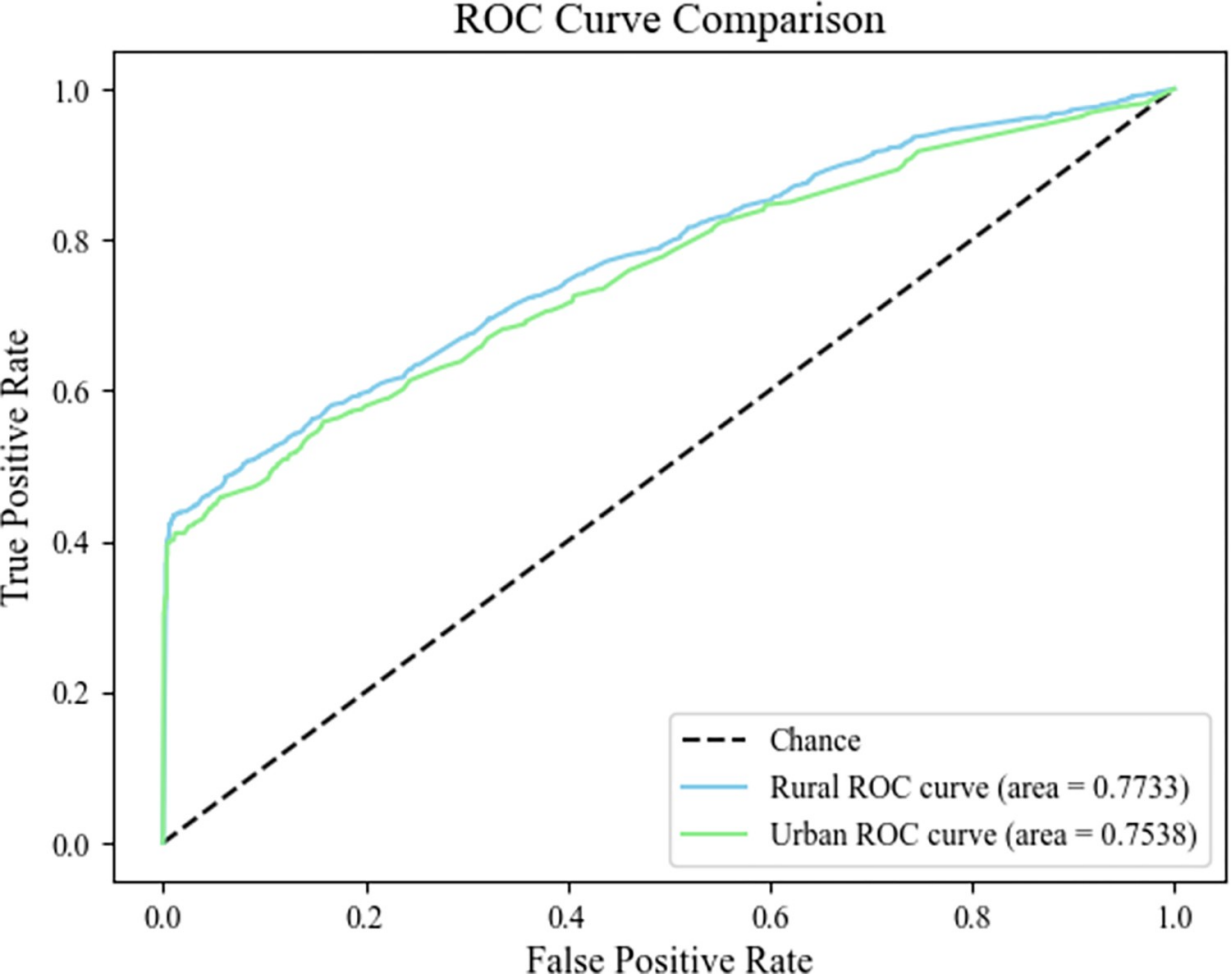
ROC curve of the random forest mode.

Stabilizing the OOB estimates for both rural and urban RF models occurs when decision trees exceed 450. This stabilization informs the construction of dysglycemia diagnostic models with accuracies of 75.35% for rural and 76.95% for urban settings. The models’ efficacy, illustrated by ROC of 0.7733 and 0.7538 respectively, is depicted in Fig 3.

## Discussion

This study investigates the prevalence and determinants of dysglycemia among urban and rural populations in Fujian Province, China, utilizing data from 26,157 adults collected through comprehensive surveys and medical examinations. The analysis reveals a dysglycemia prevalence of 35.26%, with a slightly higher rate in rural areas (35.8%) compared to urban areas (34.1%), shown in Table 1. Common influencing factors across both settings include age, BMI, hypertension, dyslipidemia, and education level. Rural-specific protective factors identified are higher income and good sleep quality, while middle income and daily sleep duration exceeding eight hours are associated with increased odds of dysglycemia. In urban areas, being male, cohabiting, and having central obesity emerge as unique factors associated with higher odds of dysglycemia. Random forest models used for predicting dysglycemia achieve accuracies of 75.35% for rural and 76.95% for urban populations, and ROC of 0.7733 and 0.7538 respectively, shown in Fig 3.

Over the past few decades, China’s economy has experienced rapid growth, albeit unevenly distributed, particularly affecting rural areas. This economic imbalance represents one of the social determinants contributing to significant health issues among the elderly population in rural China [41]. Consistent with previous surveys [42], Fujian Province in China exhibits low educational attainment with a pronounced urban-rural disparity: 45.7% of rural inhabitants and 35.2% of urban dwellers have not completed primary education (Table 1). Education lays a robust foundation for sustained cognitive growth and living conditions, offering pathways to improved employment prospects and higher income. Moreover, it stimulates the adoption of positive and healthy lifestyle choices. Collectively, these benefits are linked to a reduced likelihood of dysglycemia [43,44]. Therefore, with targeted educational initiatives, we could reduce the likelihood of urban-rural residents developing dysglycemia. Initiatives such as universities for the elderly and community health outreach programs serve as effective means to disseminate health consciousness and foundational medical knowledge [45].

Consistent with our findings, several studies have established a direct correlation between aging and the increased likelihood of developing dysglycemia. [46,47]. Age-related changes in insulin sensitivity, pancreatic β-cell function, and the accumulation of visceral fat are significant contributors to this likelihood [48]. Given the established link between aging and the heightened likelihood of dysglycemia, it becomes clear that intervention measures must pivot toward lifestyle modifications. Intervention measures should emphasize lifestyle modifications. Based on the protective factors identified through LR analysis, these include encouraging physical exercise, enhancing sleep quality, and maintaining a healthy BMI. Advancing targeted and efficient screening for dysglycemia can also contribute to increasing awareness and prevention of dysglycemia. In alignment with our observations, extensive research corroborates that hypertension and dyslipidemia predispose individuals to a heightened possibility of dysglycemia [49–52]. This concordance emphasizes the intricate interplay among these conditions, underscoring the critical need for integrated approaches in screening and managing these co-occurring health risks. Beyond the recommendations, fostering medical equity across urban and rural regions to ensure that low-income rural populations have easier access to medical and rehabilitation services is crucial. Concurrently, conducting regular health assessments for populations at risk of dysglycemia can facilitate early detection, preventing the irreversible progression to diabetes and averting severe health consequences [53,54].

This study’s findings indicate that in rural areas, higher income, good sleep quality, and high-intensity PA are protective against dysglycemia, whereas middle-income, moderate-intensity activities, and daily sleep durations exceeding eight hours are associated with an increased likelihood of dysglycemia. (Table 4). Additionally, the lower education levels and higher incidence of dyslipidemia in rural areas contribute to the higher prevalence of dysglycemia in these regions (Table 2). The farmwork demands and limited health infrastructure in rural areas further contribute to the neglect of health management and regular medical check-ups, exacerbating glycemic control issues. This also explains why the prevalence of dysglycemia is higher in rural areas of Fujian Province, China, compared to urban areas [55]. In rural settings, moderate-intensity physical activities, such as walking and labor primarily due to agricultural and occupational demands, often lack the systematic and sustained intensity found in organized high-intensity exercise, which is known to effectively reduce the likelihood of dysglycemia and provide significant metabolic benefits [56,57]. Furthermore, the dietary habits of rural inhabitants, characterized by high fat and carbohydrate intake will counteract the potential metabolic benefits of these activities, increasing the likelihood of dysglycemia [58,59]. Interestingly, the phenomenon where higher income offers protection against dysglycemia in rural residents, while middle income is associated with an increased likelihood of dysglycemia, could be attributed to lifestyle habits. Higher-income enables better access to healthcare, nutritious foods, and wellness resources, alongside more opportunities for PA and health screenings [60,61]. Conversely, middle-income individuals might afford diets high in meat and processed foods, increasing the likelihood of dysglycemia, unlike lower-income counterparts who may consume more plant-based, high-fiber diets due to financial constraints [62,63]. This scenario illustrates the intricate connections between socioeconomic status, dietary habits, and health, suggesting the importance of nuanced health interventions tailored to different income levels. Consistent with our findings, numerous studies have indicated that prolonged sleep duration (exceeding 8 hours) is associated with an increased likelihood of dysglycemia [64,65]. The link between extended sleep in rural areas and heightened dysglycemia likelihood can be attributed to multiple factors. Excessive sleeping may indicate underlying health issues, such as sleep apnea or depression, which are acknowledged risk factors for dysglycemia [66,67]. Furthermore, longer sleep durations could reflect a sedentary lifestyle, with diminished time dedicated to PA, thereby elevating the chances of dysglycemia. Concurrently, this study aligns with previous research, identifying good sleep quality as a protective factor against dysglycemia [68,69].

**Table 4:** Logistic regression results for dysglycemia (rural).

Variable	B	OR	95% CI
**Age**			
Under 65			
65 and older	0.211	1.235*	1.142–1.336
**Body mass index**			
Underweight			
Normal weight	0.112	1.119	0.951–1.316
Overweight	0.412	1.511*	1.273–1.792
Obese	0.518	1.678*	1.373–2.050
**Education**			
Below primary school			
Primary school	-0.246	0.782*	0.714–0.855
Junior high school	-0.142	0.868*	0.796–0.946
Junior college and above	-0.268	0.765*	0.680–0.848
**Annual income**			
Lower income			
Middle income	0.115	1.122*	1.033–1.219
Higher income	-0.224	0.799*	0.735–0.870
**Marital status**			
Solitary			
Cohabitation	0.018	1.018	0.926–1.119
**Daily sleep duration**			
Under 6h			
6h-8h	-0.089	0.915	0.821–1.019
Over 8h	0.136	1.146*	1.025–1.280
**Sleep quality**			
Bad			
Average	-0.114	0.892*	0.802–0.993
Good	-0.112	0.894*	0.801–0.997
**PA**			
Low PA			
Moderate PA	0.090	1.095*	1.013–1.183
High PA	-0.138	0.871*	0.788–0.963
**Chronic disease**			
None			
Yes	0.059	1.061	0.991–1.136
**Self-rated health**			
Bad			
Average	-0.005	0.995	0.889–1.114
Good	-0.121	0.886	0.784–1.001
**Hypertension**			
None			
Yes	0.424	1.528*	1.426–1.638
**Dyslipidemia**			
None			
Yes	0.441	1.555*	1.458–1.659
**Central obesity**			
None			
Yes	-0.035	0.965	0.886–1.051

* Indicates P < 0.05; The gray section: Protective factor; The blue section risk factors.

This study’s findings indicate that in urban areas, being male, cohabitation, and central obesity were identified as unique factors increasing the likelihood of dysglycemia (Table 5). Consistent with our findings, some research indicates that in urban environments, males may have a higher predisposition to dysglycemia compared to their rural counterparts and are also more susceptible to dysglycemia than urban females [70–73]. This risk factor among urban males can be attributed to lifestyle choices and occupational stressors more prevalent in urban settings, differing significantly from those in rural areas and from the lifestyle patterns of urban females. Our study found that cohabitation in urban settings increases the likelihood of dysglycemia. Numerous studies have shown that cohabitation affects individual lifestyle habits; specifically, it may lead to weight gain [74], unhealthy dietary habits [75], and reduced willingness to exercise [76,77], all of which are established risk factors for dysglycemia. Central obesity, marked by excess fat around the stomach and abdomen, is a significant risk factor for dysglycemia in urban populations [78–80]. In the surveyed urban residents, the prevalence of central obesity is notably higher at 35.0%. Therefore, the likelihood of developing dysglycemia due to central obesity is higher in urban areas [81].

**Table 5:** Logistic regression results for dysglycemia (urban).

Variable	B	OR	95% CI
**Age**			
Under 65			
65 and older	0.316	1.372*	1.211–1.555
**Gender**			
Female			
Male	0.126	1.135*	1.024–1.258
**Body mass index**			
Underweight			
Normal weight	0.178	1.195	0.917–1.556
Overweight	0.396	1.486*	1.127–1.960
Obese	0.588	1.800*	1.322–2.453
**Education**			
Below primary school			
Primary school	0.062	1.064	0.919–1.232
Junior high school	-0.078	0.925	0.802–1.067
Junior college and above	-0.204	0.815*	0.708–0.939
**Marital status**			
Solitary			
Cohabitation	0.167	1.182*	1.022–1.366
**Daily sleep duration**			
Under 6h			
6h-8h	-0.107	0.899	0.769–1.051
Over 8h	0.002	1.002	0.854–1.175
**PA**			
Low PA			
Moderate PA	-0.040	0.960	0.853–1.081
High PA	0.130	1.139	0.958–1.354
**Chronic disease**			
None			
Yes	-0.059	0.943	0.848–1.048
**Sedentary behavior**			
None			
Yes	0.068	1.070	0.961–1.192
**Self-rated health**			
Bad			
Average	0.114	1.120	0.934–1.345
Good	0.065	1.067	0.884–1.288
**Hypertension**			
None			
Yes	0.416	1.515*	1.353–1.697
**Dyslipidemia**			
None			
Yes	0.451	1.570*	1.419–1.736
**Central obesity**			
None			
Yes	0.301	1.351*	1.192–1.530

* Indicates P < 0.05; The gray section: Protective factor; The blue section risk factors.

This study evaluates the determinants of dysglycemia among residents of Fujian Province, China, employing LR to analyze their impact and developing a predictive model via an RF approach. It contributes threefold: firstly, it identifies critical factors for urban and rural dysglycemia, guiding researchers and informing policymakers on targeted prevention strategies; secondly, it assesses these factors’ influence from an urban-rural perspective, the laying groundwork for nuanced intervention in dysglycemia management; thirdly, the model enhances dysglycemia risk screening efficiency, addressing both urban and rural needs. Limitations include the inability of cross-sectional data to infer causality or dynamic variable interactions and the study’s context-specific findings to Fujian’s socio-cultural environment, which may limit broader applicability.

## Conclusions

The study, integrating logistic regression analysis and the random forest model, identified education, BMI, age, hypertension, and dyslipidemia as common factors for dysglycemia among urban and rural residents. sleep quality, PA, daily sleep duration, and annual income emerged as key factors for rural residents, while gender, marital status, and central obesity were pinpointed as specific key factors for rural inhabitants.

## Supporting information

S1 File Data cleaned. (XLSX)

S2 File Data raw. (XLSX)

S3 File Data descriptive table. (XLSX)

S4 File Code. (RAR)
